# Risk Factors of Symptomatic COVID-19 in Samtse District, Bhutan

**DOI:** 10.3389/fpubh.2022.857084

**Published:** 2022-05-02

**Authors:** Karma Lhendup, Tsheten Tsheten, Tshewang Rinzin, Kinley Wangdi

**Affiliations:** ^1^Samtse General Hospital, Samtse, Bhutan; ^2^Department of Global Health, National Centre for Epidemiology and Population Health, Australian National University, Canberra, ACT, Australia; ^3^Royal Centre for Disease Control, Ministry of Health, Thimphu, Bhutan

**Keywords:** COVID-19, Bhutan, symptomatic, risk factors, survival analysis, Samtse District, quarantine

## Abstract

**Introduction:**

All Coronavirus disease 2019 (COVID-19) positive cases in Samtse District, Bhutan were isolated in the isolation facilities managed by the government hospitals. This study aimed to identify the socio-demographic risk factors for developing COVID-19 symptoms amongst these patients.

**Methods and Materials:**

A secondary data of the COVID-19 positive cases from isolation facilities of Samtse District from 5 May to 7 September 2021 was used for this study. Survival analysis was carried out to estimate the cumulative probability of symptom onset time by each risk factor. Kaplan–Meier curves were used to estimate the probabilities for the onset of symptoms at different time points and a log-rank test was employed to assess the differences between covariates.

**Results:**

A total of 449 patients were included, of which 55.2% were males and 73.3% (328) were aged >18 years. The mean age was 42 years with a range of 3 months to 83 years. Forty-seven percent (213) reported at least one symptom. Common symptoms were fever (32.3%, 145), headache (31.6%, 142), and cough (30.1%, 135), respectively. Males were 64% less likely to be symptomatic than females [adjusted hazard ratio (aHR) = 0.36, 95% confidence interval (CI) 0.183–0.917]. Farmers (aHR = 3.17, 95% CI 1.119–8.953), and drivers and loaders (aHR = 3.18, 95% CI 1.029–9.834) were 3 times more likely to be symptomatic compared to housewives. Residents of Samtse sub-districts were 5 times more likely to be symptomatic than those living in other sub-districts (aHR = 5.16, 95% CI 2.362–11.254).

**Conclusion:**

The risk of developing COVID-19 symptoms was being fe male, farmers, drivers and loaders, and residents of the Samtse sub-district. These high-risk groups should be provided additional care when in isolation facilities.

## Introduction

A cluster of patients with severe pneumonia of unknown etiology appeared in China in December 2019, leading to the discovery of a novel coronavirus—severe acute respiratory syndrome coronavirus 2 (SARS-CoV-2) (1). The World Health Organization (WHO) later renamed the disease Coronavirus disease 2019 (COVID-19) in February 2020 (2). Due to the worldwide spread of the disease, the WHO declared the event a public health emergency of international concern in January 2020 (3). As of 7 January 2022, 298,915,721 cases and 5,469,303 confirmed deaths were reported globally (4). Despite the worldwide rollout of vaccines, newer strains such as Delta and Omicron have emerged, resulting in new waves of COVID-19 infections (5–13).

Bhutan reported its first case of COVID-19 on 5 March 2020, which was confirmed in an American tourist (14). However, no transmission in the community was reported for 13 months due to successful public health interventions. These included quarantining all incoming travelers for 21 days (15). All non-essential activities were halted and a social distance of 1.5 m and a face mask in public places were made mandatory (15). Flu clinics were set up across the country so that people did not have to visit hospitals for flu-like symptoms, thereby reducing the risk of transmission to people visiting hospitals. All patients needing admission to the hospital were tested for COVID-19. Towns and districts along the Indian border have been identified as Red Zone and a 7-day mandatory quarantine is required for people traveling out of these areas.

The first community transmission was officially reported in Phuntsholing Municipality under Chukha District on 16 April 2021. Since then Bhutan saw several community transmissions in various parts of the country mostly in the southern regions which share an international border with India. Bhutan has continued to close its borders with India and restrict the movement of non-essential goods and people since 23 March 2020 (16, 17). As of 24 March 2022, Bhutan had a total of 21,660 cases with only nine deaths (18).

Samtse District, located in South West of Bhutan, shares a long and porous border with India. As a result, people living in Samtse District are highly vulnerable and at risk of COVID-19 infection. Therefore, Samtse is one of the Red Zone districts in Bhutan. The first case of COVID-19 in the district was reported on 20 July 2020 from a quarantine facility in an expatriate worker from India. While the first community transmission of COVID-19 was reported on 9 May 2021 in an individual working at the border guarding the Point of Entry (POE). The community transmission continued for 3 months and recorded a total of 449 cases. All positive patients confirmed by reverse transcription-polymerase chain reaction (RT-PCR) were isolated in the Isolation facilities to monitor and manage COVID-19 symptoms and prevent the spread of the SARS-CoV-2. They were re-tested on the 22^nd^ day and discharged once the results were negative.

COVID-19 has a wide range of clinical manifestations which vary from highly subclinical infection to pneumonia resulting in deaths in some cases (19, 20). Asymptomatic individuals have a substantial role in the spread of SARS-CoV-2 to healthy individuals for up to 2 weeks (21, 22). The risk of the onset of symptoms, hospitalizations, and mortalities depends on demographic characteristics, underlying medical conditions, and vaccination status (23). Hence, understanding the clinical and demographic characteristics of COVID-19 and identifying risk factors for symptomatic COVID-19 will be useful for future prevention and management of cases. However, there are limited studies on the clinical manifestations and their associated risk in Bhutan (24). The findings from this study will be useful in addressing this gap and provide evidence for the region-specific COVID-19 disease profile. In addition, the findings can be used to inform differential and coordinated district-specific responses. This study aimed to describe the demographic and clinical characteristics of COVID-19 and identify the risk factors of symptomatic COVID-19 in the Samtse District.

## Materials and Methods

### Study Design and Setting

**Figure 1:**
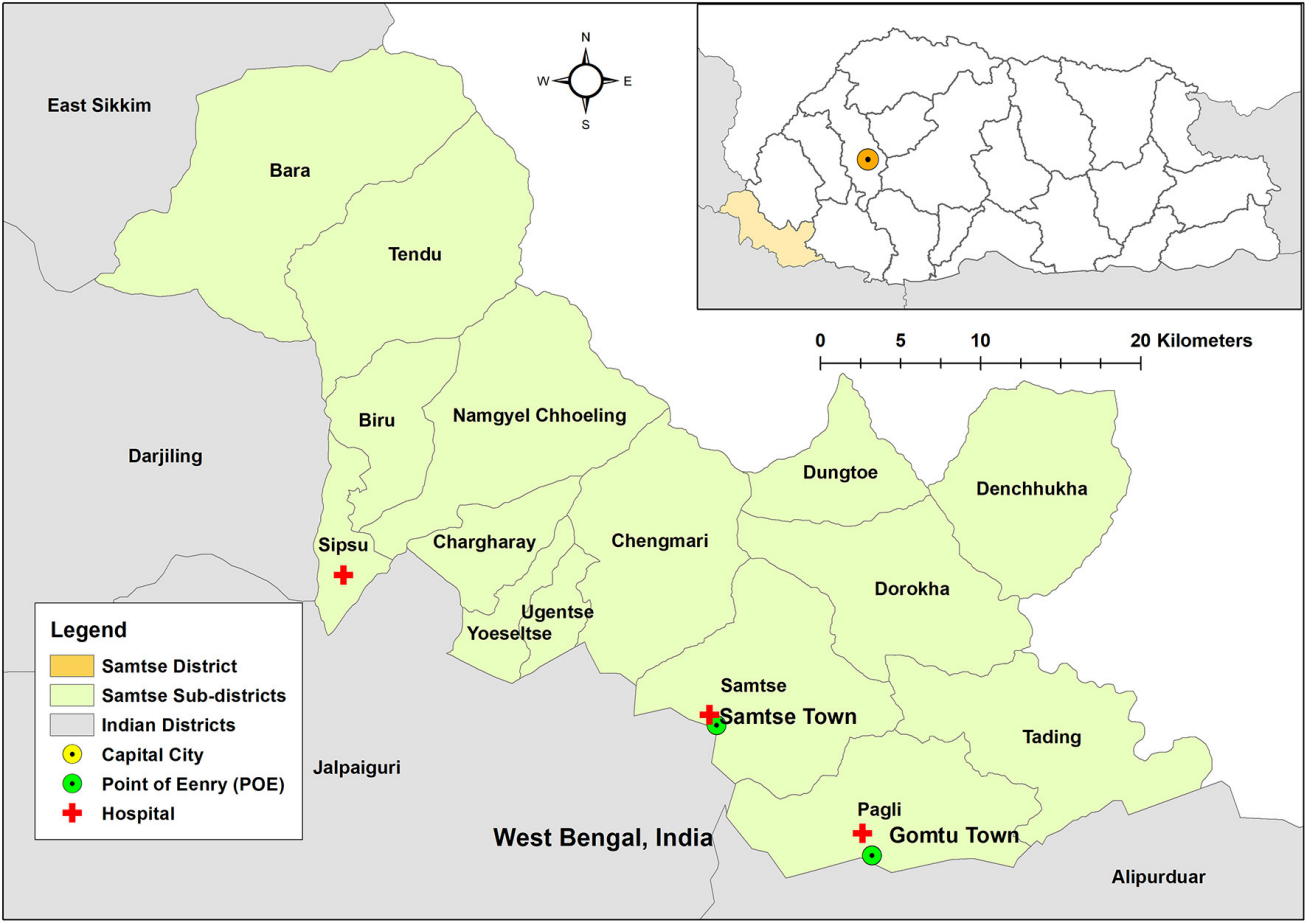
Samtse District (study area) and its location, Bhutan.

This was a retrospective study using the secondary data of COVID-19 cases in the Samtse District. Samtse shares borders with the Indian state of West Bengal in the south, Kalimpong, and Sikkim in the west (Figure 1). In 2017, the total population of Samtse District was 62,787, consisting of 32,022 males and 30,568 females (25). Administratively, Samtse District is divided into 15 sub-districts or *Gewogs*. The most populace sub-district was Tendruk with 6,242 people. There are three hospitals (Samtse, Sibsoo, and Gomtu), 12 Primary Health Centre, and five sub posts in the district. All three hospitals have facilities for patient admission and Samtse General Hospital being the district hospital is 40 bedded and provides specialized services including Obstetrics & Gynecology, Pediatrics, Medical specialists, and surgical. All COVID-19 cases in the district were managed by three hospitals. A separate flu clinic was set up in the catchment areas of these hospitals. Five medical officers (MOs) have been identified for COVID-19 duty (Samtse Hospital- 3 MOs, and one each for Sibsoo and Gomtu hospitals, respectively). A total of 40 nurses (in all three hospitals) were available for the management of patients with COVID-19, and this clinical staff was trained in the management of COVID-19 cases.

### Study Population

The study population included all COVID-19 positive individuals from the Isolation facilities of Samtse District from 5 May to 7 September 2021. The three isolation facilities were Samtse, Gomtu, and Sibsoo hospitals. COVID-19 cases included those frontline workers, people from the community who tested positive on routine testing, and also those who turned positive on RT-PCR during 21 days of quarantine. In addition, positive cases from the flu clinics, contact tracing following an index case of COVID-19 in the community, and mass community testing were also included in this study. The COVID-19 samples were shipped to Phuntsholing COVID-19 Laboratory (located 80 km away from Samtse) since the RT-PCR facility was not available in Samtse. The nurses on duty recorded the signs and symptoms of the patients during their admission or as and when patients reported.

Inclusion criteria: (i) all RT-PCR positive patients, (ii) all ages, (iii) both sexes, (iv) admitted to COVID-19 isolation wards of Samtse, Sibsoo, and Gomtu, and (v) complete information on dates of admission, symptom onset, and date discharge.

Exclusion criteria: (i) incomplete patient information, (ii) no admission date/date of discharge, (iii) no symptom onset date, (iv) missing sociodemographic characteristics.

### Data Collection

The details of the COVID-19 cases were extracted from the admission registers maintained by the Isolation facilities of the three hospitals (Samtse, Gomtu, and Sibsoo). The following information was extracted: age, sex, area of residence, symptoms, comorbidity, date of symptom onset, place of detection, vaccination status, and date of discharge. The data extraction form has been attached (Supplementary Table 1). An electronic map of Samtse District in shapefile format was obtained from the DIVA-GIS database (https://www.diva-gis.org/).

### Statistical Analysis

Descriptive analysis in terms of frequencies and percentages was used to present the socio-demographic and clinical characteristics of the study participants. Independent variables included demographic characteristics (age, sex, education level, occupation), vaccination status, and co-morbidities, while the symptomatic/asymptomatic COVID-19 comprised the dependent variable.

Survival analysis was undertaken with symptomatic cases as the primary outcome; defined as the manifestation of symptoms in an RT-PCR confirmed SARS-CoV-2 infection. For the survival analysis, only those who developed symptoms while in the isolation were included. Following the onset of symptoms, they were censored meaning their symptom-free time ended.

A total of 211 records were deleted from survival analysis because they developed symptoms on or before admission. Further two records were removed because they spent >21 days in the quarantine. Cox proportional hazards regressions were used to compute hazard ratios (HR) associated with demography, vaccination, and co-morbidities characteristics. A statistically significant HR was indicated by 95% confidence intervals (CI) excluding 1. An α-level of 0.05 was also used to indicate the statistical significance of all analyses.

The data were analyzed using STATA version 16 (Stata Corporation, College Station, TX, USA) software. Maps were produced using ArcMap 15.1 (ESRI, Redlands, CA).

## Results

### Participant Characteristics

**Figure 2:**
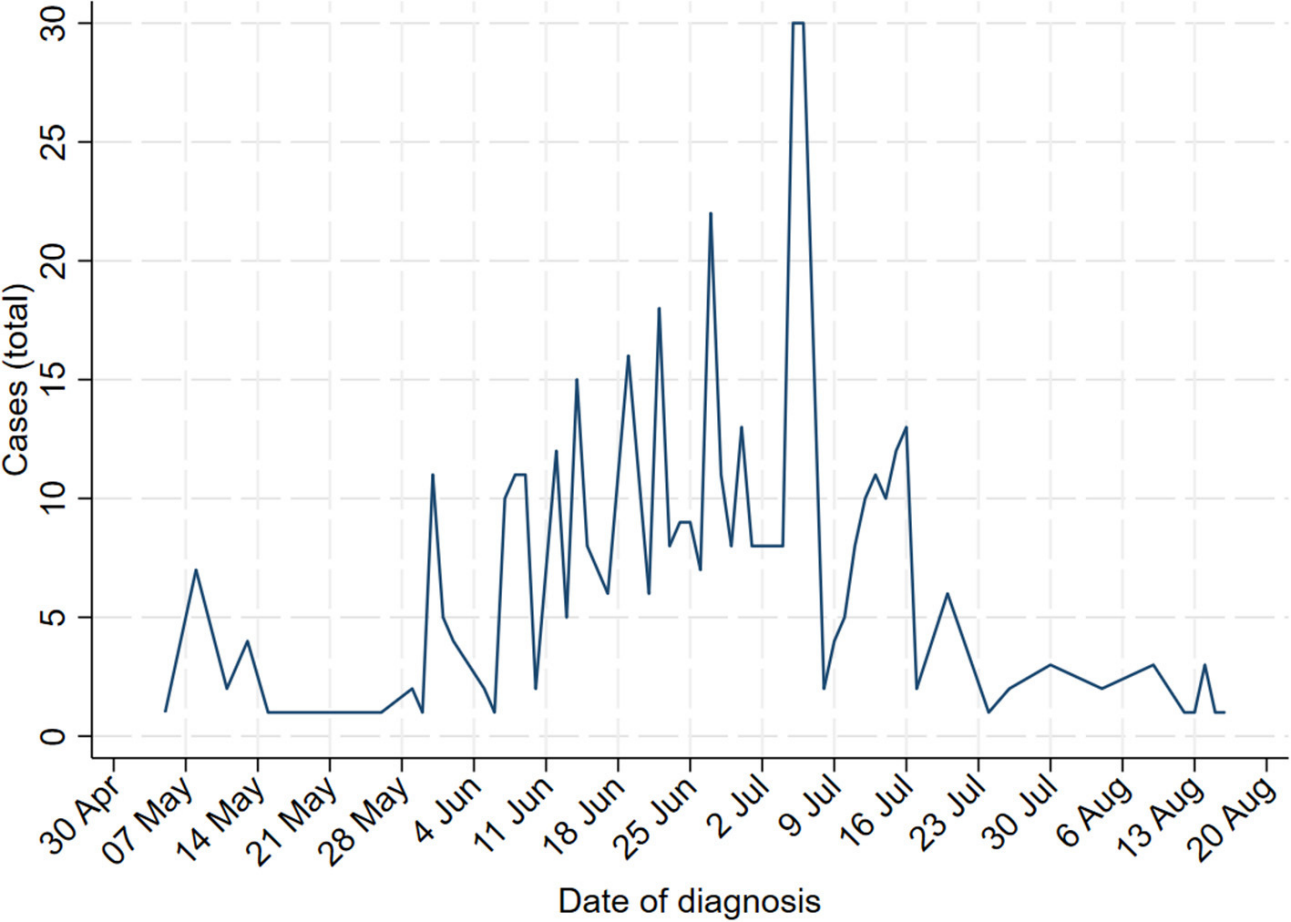
Date of diagnosis of COVID-19 cases in Samtse District.

A total of 449 participants were included in the study from six sub-districts in Samtse District. COVID-19 cases were reported between 5 May to 7 September 2021 (Figure 2). Males were slightly higher than females (males vs. females: 55.2 vs. 44.8%). The mean age of the patients was 42 years (ranging from 3 months to 83 years). Participants aged >18 years were proportionately higher with 73.3% (*n* = 328), while those ≤ 18 years were 27.0% (*n* = 121). While both passive and active case investigations were used to recruit the study participants, active case investigations detected a predominantly higher number of cases (passive vs. active: 10.5 vs. 89.5%). The vaccinated population accounted for 73.0% (*n* = 328) of the total participants. The commonest occupations were office goers (28.0%, *n* = 130), housewives (13.1%, *n* = 59), and farmers (9.8%, *n* = 44). Children or students made up 35.9% (*n* = 161) of the study population. Residents of Samtse sub-district made up 55.9% (*n* = 251) of cases. An 82 old woman died and another patient was referred to as the patient attendant of this patient (Table 1). Eleven individuals reported one or more co-morbidities, hypertension (*n* = 5) being the commonest co-morbidity (Supplementary Table 1).

**Table 1:** Demographic characteristics of the laboratory-confirmed COVID-19 in Samtse District, Bhutan.

**Variables**		**Total** **(*****N*** **=** **445)**		**Symptomatic** **(*****n*** **=** **261)**	
		**Number**	**Percent**	**Number**	**Percent**
**Sex**					
	Female	201	44.8	121	45.8
	Male	248	55.2	143	54.2
**Age**					
	≤ 18	121	27.0	57	21.6
	>18	328	73.0	207	78.4
**Occupation**					
	Housewife	59	13.1	33	12.5
	Office goers	130	28.0	93	35.2
	Children/student	161	35.9	81	30.7
	Farmers	44	9.8	24	9.2
	AFS	15	3.3	12	4.6
	DL	40	8.9	21	8.0
**Vaccination**					
	No	121	27.0	55	20.8
	Yes	328	73.0	209	78.2
**Detection mode**					
	Health facilities (passive)	47	10.5	37	14.0
	Active case investigation	402	89.5	227	65.0
**Sub-districts**					
	Other	198	44.1	90	34.1
	Samtse	251	55.9	174	65.9
**Outcome**					
	Recovered	447	99.6		
	Died	1	0.2		
	Referred	1	0.2		

*AFS, armed forces and Desuups; DL, Drivers and loaders*.

**Figure 3:**
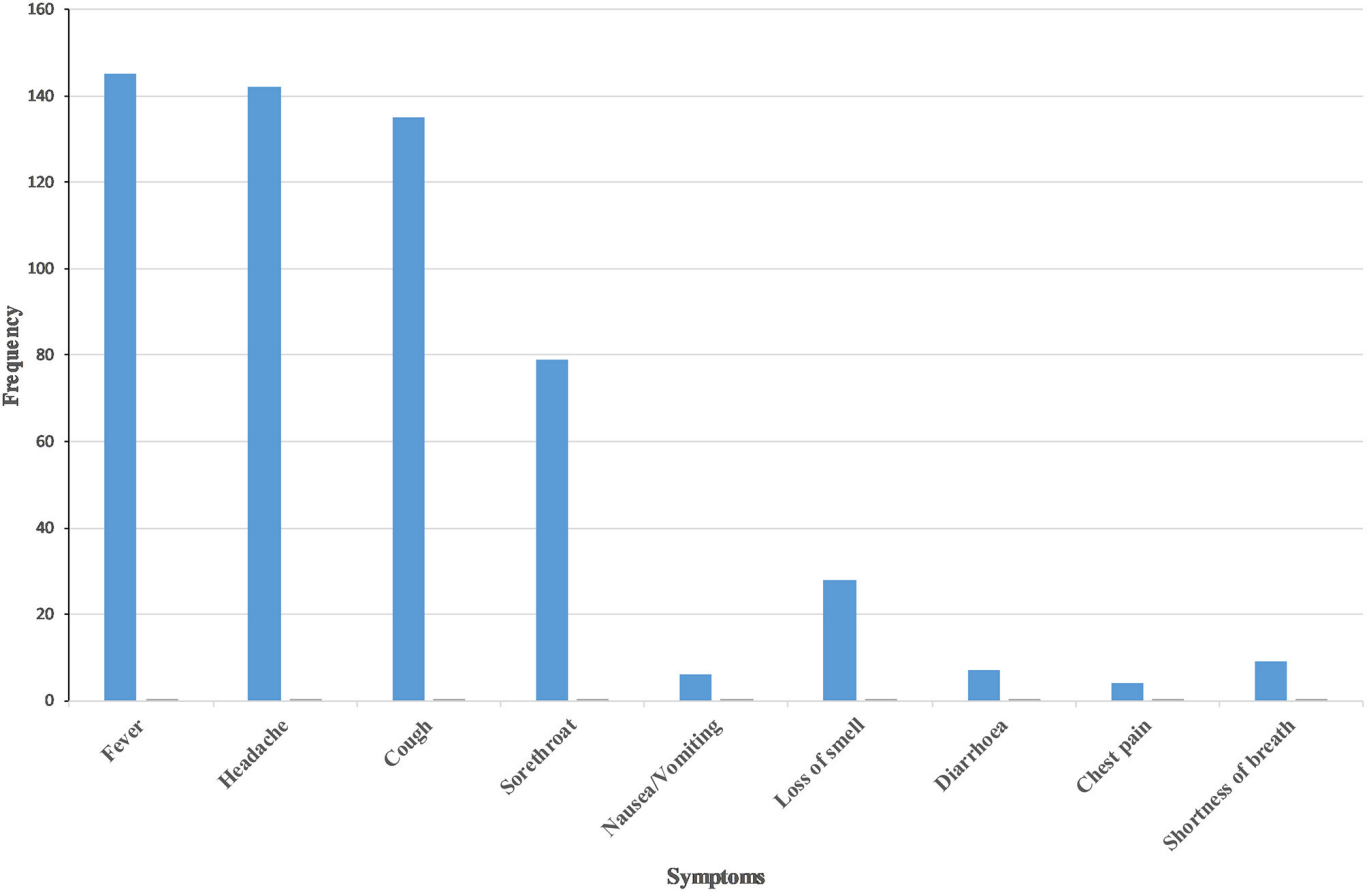
Clinical features of COVID-19 in Samtse District, Bhutan.

The commonest COVID-19 symptom was fever (32.3%, *n* = 145) followed by headache (31.6%, *n* = 142) and cough (30.1%, *n* = 135). Sore throat (17.5%. *n* = 79) and loss of smell (6.3%, *n* = 28) were infrequently reported (Figure 3). Nearly half (47.4%, *n* = 213) reported symptoms either before or on the day of admission. Two cases were admitted for more than 21 days (results not shown).

### Survival Analysis

**Figure 4:**
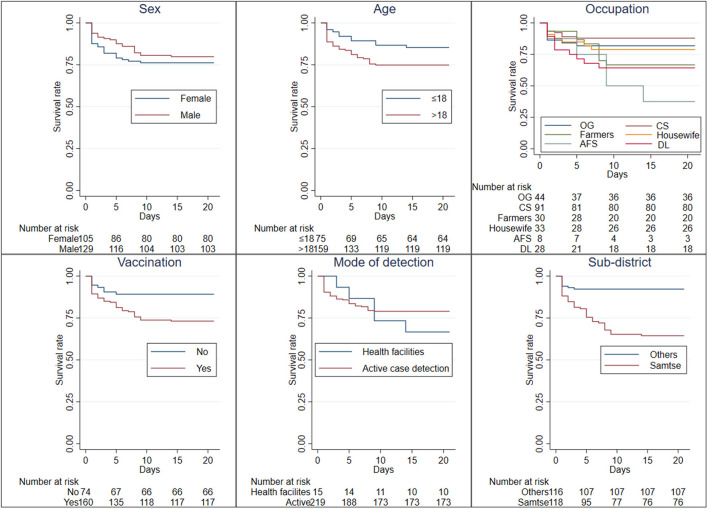
Kaplan-Meier survival estimates for the probability of developing symptomatic COVID-19 in Samtse District, Bhutan. OG, office goers; CS, children/students; AFS, Armed forces and *Desuups*; DL, drivers and loaders.

Kaplan–Meier curves revealed a significant difference in cumulative survival for occupation (chi-square =16.95, *p* = 0.0046), vaccination status (chi-square = 7.13, *p* = 0.0076), and residents of Samtse sub-districts (chi-square = 25.41, *p* < 0.001) (Figure 4, Supplementary Table 2). Cox proportional hazard analysis showed that males were 64% less likely to be symptomatic than females [adjusted hazard ratio (aHR) = 0.36, 95%, confidence interval (CI) 0.183–0.917]. Farmers (aHR = 3.17, 95% CI 1.119–8.953), and drivers and loaders (aHR = 3.18, 95% CI 1.029–9.834) were three times more likely to be symptomatic compared to housewives. Residents of Samtse sub-districts were 5 times more likely to be symptomatic than those living in other sub-districts (aHR = 5.16, 95% CI 2.362–11.254). However, age, mode of detection, and vaccination were not associated with developing symptoms (Table 2).

**Table 2:** Determinants of COVID-19 symptoms using Cox proportional hazard analysis, Samtse District, Bhutan.

	**Variables**	**aHR**	**95 % CI**	***p*** **-values**
**Sex**				
	Female	Ref		
	Male	0.36	0.183–0.917	0.004
**Age**				
	≤ 18	Ref		
	>18	0.44	0.106–1.3823	0.258
**Occupation**				
	Housewife	Ref		
	Office goers	1.58	0.539–4.728	0.412
	Children/student	1.64	0.431–6.227	0.468
	Farmers	3.17	1.119–8.953	0.03
	AFS	2.67	0.569–12.559	0.213
	DL	3.18	1.029–9.834	0.044
**Vaccination**				
	No	Ref		
	Yes	3.65	0.827–16.120	0.087
**Detection mode**				
	Health facilities	Ref		
	Active case investigation	1.15	0.293–4.541	0.2
**Sub-districts**				
	Others	Ref		
	Samtse	5.16	2.362–11.254	<0.001

*aHR, adjusted hazard ratio; CI, confidence interval; AFS, armed forces and Desuups; DL, Drivers and loaders*.

## Discussion

After the first case of COVID-19 in the community on 11 May 2021, community transmission continued for 3 months with a total of 449 cases from six sub-districts of Samtse District. COVID-19 was predominantly among males, >18 years and residing in Samtse Sub-district. The most common symptoms among the study participants were fever, headache, and cough. One COVID-19 death was reported in a patient detected in Samtse. Symptomatic COVID-19 was associated with being male, working as farmers, driver and loader, and residents of the Samtse sub-district.

An 82 years old woman died while in COVID-19 isolation in Phuentsholing Hospital. She was referred from the Sibsoo Isolation facility to Phuntsholing Hospital due to deteriorating condition requiring ventilation which was available only in Phuntsholing Hospital. The patient had many co-morbidities including hypertension, chronic obstructive pulmonary disease (COPD), and heart failure. The risk of death in patients with co-morbidities has been documented (26, 27).

In this study, the common symptoms were fever, cough, and headache. These findings were in agreement with other studies, where commonly reported symptoms were fever, sore throat, headache, cough, and myalgia (19, 24, 28). COVID-19 tends to be more severe in adults and the elderly with devastating health outcomes in immunocompromised and patients with co-morbidities (26, 29). However, in this study, age was not associated with developing symptoms. This can be partly explained by the absence of co-morbidities in most patients with only 11 patients reporting one or more comorbidities.

Males were less likely to develop COVID-19 symptoms as compared to females. This is different from the findings of a systematic review, where men were more likely to develop symptoms (30) that were attributed to risky behavior including smoking and alcohol use in men (31–33). The higher risk of symptomatic COVID-19 in women in our study might be related to different socio-demographic characteristics such as the health-seeking behavior of females, which tends to be higher than males (34). Hence, the study underscores the paramount importance of recognizing the location-specific risk factors for COVID-19.

Farmers, drivers, and loaders were the occupational groups associated with an increased risk of developing COVID-19 symptoms. Some occupation groups are at a greater risk of COVID-19 infection (35–37). Farmers make up a large proportion of the rural population in Bhutan. Many villages in Samtse District are located along the international border in proximity to Indian villages. A study in the US found that the farmers were at higher risk of contracting COVID-19 (38). This has led to a reduction in farm produce across the world (39, 40). This is difficult to ascertain in Bhutan due to a lack of data. Therefore, it is important to consider this issue in future studies as the pandemic continues with newer variants.

In addition, drivers and loaders were also found to be significantly associated with COVID-19 symptoms. A study on the work-related COVID-19 in six Asian countries including Hong Kong, Japan, Singapore, Taiwan, Thailand, and Vietnam reported “drivers and transport workers” contributed upto 18% of the COVID-19 burden (37). A similar finding of a higher risk of reporting symptoms among people working in the transportation services was also reported in Phuntsholing Municipality in Bhutan (24). Loaders tranship the goods entering Bhutan, from Indian vehicles to Bhutanese vehicles. Even though there is a non-contact protocol in place, where Indian transporters including drivers and their helpers park their vehicles and stay in the isolation quarters set up for this purpose. In addition, the surface of vehicles is disinfected before transshipment can begin. All loaders are provided with standard personal protective equipment (PPE) and are made to stay in a separate place isolated from other populations including their families. Despite these protocols, the first case of COVID-19 from the community in Bhutan was reported among people working in mini-dry project in Phuntsholing. Recognizing the risk associated with this occupation, the Bhutan government has instituted mandatory testing of all frontline workers (41).

The people in Samtse sub-district were at a higher risk of developing symptoms as compared to other districts. Samtse District is located in the southwest of Bhutan and shares a long stretch of borders with the Indian states of Alipurduar and Jalpaiguri District of West Bengal in the south and Kalimpong and Sikkim in the west. For this reason, Samtse District falls under the COVID-19 Red Zone in Bhutan. All forms of non-emergency movements from the Red Zone to other districts are restricted. People have to undergo 1 week of mandatory facility quarantine before moving from Red Zone districts to other districts. Samtse District is divided into 15 sub-districts and Samtse Town is the headquarters of the Samtse District located in Samtse sub-district. During the pandemic, all POE from India into Samtse District has been closed except Samtse and Gomtu towns. Therefore, all movements of goods and people into Samtse District enter from these POEs. However, people from India and other parts of Bhutan transit to Samtse District through Samtse Town. As a result, residents of Samtse Town which is located in the Samtse sub-district are at a higher risk of COVID-19 infection (Figure 1). This is evident from this study where 55.9% of cases were from the Samtse sub-district.

There are a number of limitations to this study. This is a retrospective study based on limited data available in the isolation facilities. Refined analyses and better insights could have been possible if more nuanced information on laboratory parameters were available. Secondly, the onset of symptoms could be subjected to the infection with different variants of SARS-CoV-2. Due to the limited testing facilities, different types of variants could not be elicited. Although a complete epidemiological profile of COVID-19 can be drawn up at the end of the epidemic, the findings from this study will adequately inform differential and coordinated district-specific responses for health resource planning and resource allocation. Third, people with co-morbidity are more likely to have poor outcomes (42, 43), which could be due to less number of co-morbidities in patients with COVID-19 (11 cases); this variable could not be included in the survival analysis. Notwithstanding these limitations, the present study is an attempt to derive meaningful information from the available data in Samtse District.

## Conclusion

In this study, we identified the socio-demographic factors that increase the risk of developing COVID-19 symptoms in a SARS-CoV-2 infection. Females, farmers, drivers, and loaders were at an increased risk of developing symptoms of COVID-19. People residing in Samtse sub-district were more likely to develop symptoms. Additional support and care should be provided to these high-risk groups during admission and while in the isolation. Public health officials should target these groups for health education and reinforce the use of personal protection including using a face mask, hand hygiene, and social distancing.

## Ethics Considerations

The administrative clearance to use this data has been approved by the Ministry of Health, Bhutan vide letter number (PPD/admin. CI/(9)/2020-21/182 dated 6 October 2021). Ethical approval was provided by the Research Ethics Board of Health (REBH), Ministry of Health, Bhutan (Ref no REBH/Approval/2021/135). Permission was approved by the Samtse District Health Sector, hospital administrators, and Medical officer Incharges to use these datasets.

## Data Availability Statement

The data analyzed in this study is subject to the following licenses/restrictions: Bhutan Ministry of Health owns the data. Requests to access these datasets should be directed to karyang4@gmail.com.

## Ethics Statement

The studies involving human participants were reviewed and approved by Research Ethics Board of Health (REBH), Ministry of Health, Bhutan.

## Author Contributions

KL and KW were involved in the conception, design of this study, and provided a critical revision of the manuscript. KL and TR undertook collection and interpretation of results and drafted the manuscript. TT and KW undertook analysis and interpretation of the results. All authors read and approved the final manuscript.

## Funding

KW was funded by Australian National Health and Medical Research Council Investigator Grant (2008697).

## Conflict of Interest

The authors declare that the research was conducted in the absence of any commercial or financial relationships that could be construed as a potential conflict of interest.

## Publisher's Note

All claims expressed in this article are solely those of the authors and do not necessarily represent those of their affiliated organizations, or those of the publisher, the editors and the reviewers. Any product that may be evaluated in this article, or claim that may be made by its manufacturer, is not guaranteed or endorsed by the publisher.
