# Supplementary material for: Risk Factors of Symptomatic COVID-19 in Samtse District, Bhutan
Source: Front Public Health. 2022 May 2;10:857084. doi: 10.3389/fpubh.2022.857084 (PMC9108283; doi:10.3389/fpubh.2022.857084)
Supplement: Supplementary file 1 [file Data_Sheet_1.docx]

**Supplementary material**

**Supplementary figure 1 Forest plot for Hazard Ratios and 95% confidence intervals of Cox proportional hazard analysis, Samtse District, Bhutan.**


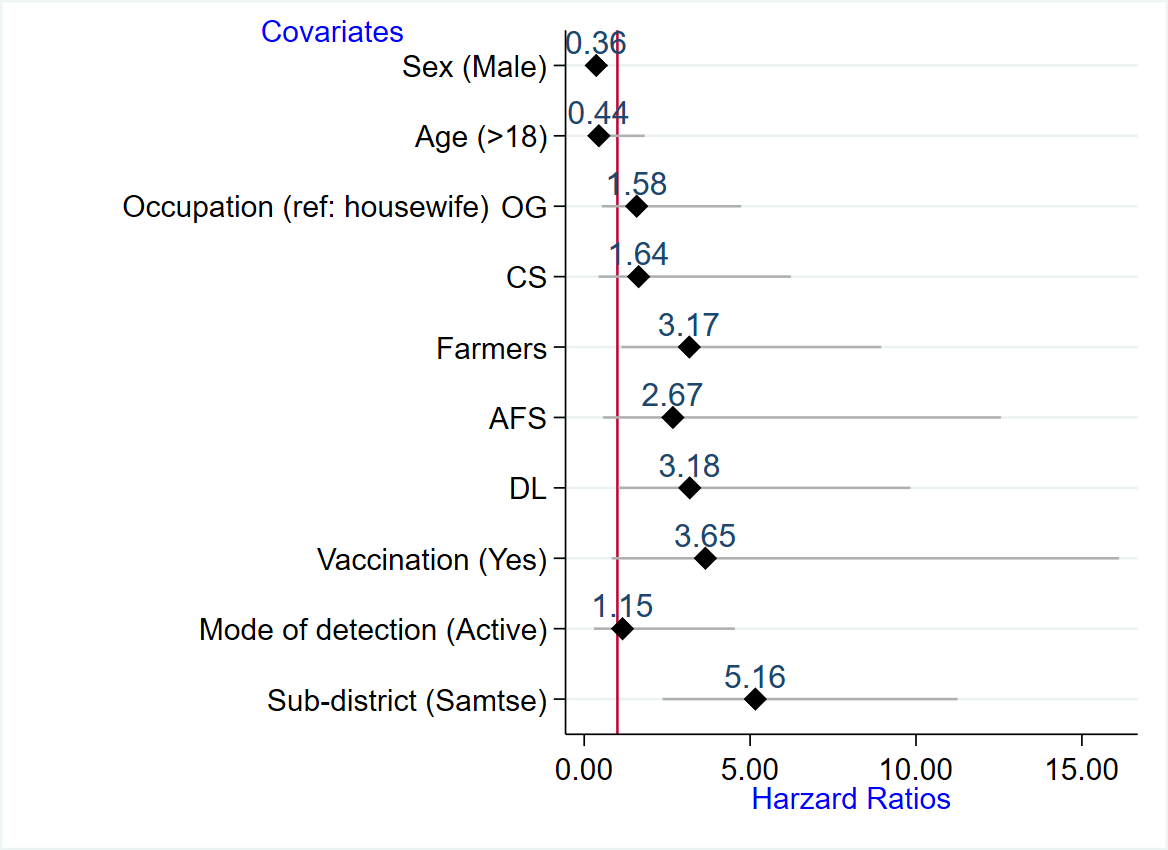


**Supplementary Table 1. Comorbidities among the COVID-19 patients in Samtse District, Bhutan.**

| **Co-morbidities** | **Number** | **Percent** |
| --- | --- | --- |
| Asthma | 1 | 0.2 |
| Diabetes mellitus | 1 | 0.2 |
| Diabetes mellitus and Hypertension | 2 | 0.4 |
| Hypertension | 5 | 1.1 |
| Anxiety disorder | 1 | 0.2 |
| Diabetes mellitus, Hypertension and heart failure | 1 | 0.2 |
| No comorbidity | 438 | 97.5 |

**Supplementary Table 2. Log-rank test for Kaplan–Meier curves**

| Variables | Chi-Square | p-value |
| --- | --- | --- |
| Sex | 0.63 | 0.4289 |
| Age | 3.39 | 0.0656 |
| Occupation | 16.95 | 0.0046 |
| Vaccination | 7.13 | 0.0076 |
| Detection mode | 0.82 | 0.3644 |
| Sub-districts | 25.41 | <0.001 |
